# Chronic Venous Disease in Pregnant Women Causes an Increase in ILK in the Placental Villi Associated with a Decrease in E-Cadherin

**DOI:** 10.3390/jpm12020277

**Published:** 2022-02-14

**Authors:** Miguel A. Ortega, Chen Chaowen, Oscar Fraile-Martinez, Cielo García-Montero, Miguel A. Saez, Iris Cruza, Claude Pereda-Cerquella, Miguel Angel Alvarez-Mon, Luis G. Guijarro, Yuliia Fatych, César Menor-Salván, Melchor Alvarez-Mon, Juan De Leon-Luis, Julia Buján, Natalio Garcia-Honduvilla, Coral Bravo, Angel Asúnsolo-del-Barco

**Affiliations:** 1 Department of Medicine and Medical Specialties, Faculty of Medicine and Health Sciences, University of Alcalá, 28801 Alcalá de Henares, Spain; oscar.fraile@edu.uah.es (O.F.-M.); cielo.garciam@edu.uah.es (C.G.-M.); msaega1@oc.mde.es (M.A.S.); iri.cruza@edu.uah.es (I.C.); claude.pereda@edu.uah.es (C.P.-C.); miguelangel.alvarezm@edu.uah.es (M.A.A.-M.); mademons@gmail.com (M.A.-M.); mjulia.bujan@uah.es (J.B.); natalio.garcia@uah.es (N.G.-H.); 2 Ramón y Cajal Institute of Healthcare Research (IRYCIS), 28001 Madrid, Spain; luis.gonzalez@uah.es (L.G.G.); angel.asunsolo@uah.es (A.A.-d.-B.); 3 Cancer Registry and Pathology Department, Hospital Universitario Principe de Asturias, 28801 Alcalá de Henares, Spain; 4 Department of Surgery, Medical and Social Sciences, Faculty of Medicine and Health Sciences, University of Alcalá, 28801 Alcalá de Henares, Spain; chen.chaowen@edu.uah.es; 5 Pathological Anatomy Service, Central University Hospital of Defence-UAH, 28001 Madrid, Spain; 6 Unit of Biochemistry and Molecular Biology (CIBEREHD), Department of System Biology, University of Alcalá, 28801 Alcalá de Henares, Spain; yuliia.fatych@edu.uah.es (Y.F.); cesar.menor@uah.es (C.M.-S.); 7 Immune System Diseases-Rheumatology and Oncology Service, University Hospital Príncipe de Asturias, CIBEREHD, 28801 Alcalá de Henares, Spain; 8 Department of Public and Maternal and Child Health, School of Medicine, Complutense University of Madrid, 28040 Madrid, Spain; jaleon@ucm.es; 9 Department of Obstetrics and Gynecology, University Hospital Gregorio Marañón, 28009 Madrid, Spain; 10 Health Research Institute Gregorio Marañón, 28009 Madrid, Spain

**Keywords:** chronic venous disease (CVD), pregnancy, integrin-linked kinase (ILK), cadherins, cell behavior, extracellular matrix (ECM)

## Abstract

Chronic venous disease (CVD) is a multifactorial vascular disorder frequently manifested in lower limbs in the form of varicose veins (VVs). Women are a vulnerable population for suffering from CVD, especially during pregnancy, when a plethora of changes occur in their cardiovascular system. Previous studies have indicated a worrisome association between CVD in pregnancy with the placental structure and function. Findings include an altered cellular behavior and extracellular matrix (ECM) composition. Integrin-linked kinase (ILK) is a critical molecule involved in multiple physiological and pathological conditions, and together with cadherins, is essential to mediate cell to ECM and cell to cell interplay, respectively. Thus, the aim of this study was to evaluate the implication of ILK and a set of cadherins (e-cadherin, cadherin-6 and cadherin-17) in placentas of women with CVD in order to unravel the possible pathophysiological role of these components. Gene expression (RT-qPCR) and protein expression (immunohistochemistry) studies were performed. Our results show a significant increase in the gene and protein expression of ILK, cadherin-6 and cadherin-17 and a decrease of e-cadherin in the placenta of women with CVD. Overall, this work shows that an abnormal expression of ILK, e-cadherin, cadherin-6 and cadherin-17 may be implicated in the pathological changes occurring in the placental tissue. Further studies should be conducted to determine the possible associations of these changes with maternal and fetal well-being.

## 1. Introduction

Chronic Venous Disease (CVD) is a vascular disorder frequently manifested by the appearance of varicose veins (VVs), generally in the lower limbs [1]. Women seems to be more prone to develop CVD, especially during pregnancy, as the whole organism undergoes a plethora of physiological, anatomical and adaptative changes which are critical for the fetus [2]. In the cardiovascular system, these alterations include notable hemodynamic modifications, variations in the oxygen transport and a profound vascular remodeling [3,4]. Occasionally, this may lead to the development of CVD, which is associated with the detection of different local and systemic markers of damage that could entail negative consequences for the maternofetal well-being [5]. Indeed, different studies indicate that approximately 1 in 3 women during pregnancy may suffer from VVs [6,7] and these data could increase to 50–70% when considering additional manifestations of CVD [8]. Thus, further studies are needed to understand the complex implications of pregnancy-related CVD for both women and fetus in order to achieve a better management of such a common condition.

CVD is also recognized as venous dysfunction. Its analogue in the arterial system, pre-eclampsia, is one of the most worrisome complications of pregnancy [9]. Previous studies have demonstrated that the placenta is the hardest-hit organ affected by this malady, defining a set of abnormal processes and markers of damage in this structure [10,11,12]. In this line, we have demonstrated that maternal venous dysfunction also induces multiple abnormalities and damage in the placenta, evidencing an increase of hypoxic markers and enhanced apoptosis [13], markers of oxidative stress [14] and altered angiogenesis and lymphangiogenesis [15]. Moreover, we also found that CVD is associated with changes in the placental composition [7,16] and signaling [17], suggesting that this condition drives detrimental modifications in the placental structure and functioning, probably representing a unique pathophysiological feature in response to the venous dysfunction.

In this context, the study of the cellular transduction of external signals may be of great aid to understand the pathophysiological mechanisms of CVD in the placenta. Integrin-linked kinase (ILK) is an intracellular molecule that binds to the cytoplasmic domain of β1 and β3-integrin, and it is considered a crucial mediator of the cell-ECM interactions [18]. The relevance of this component has been widely established in the cardiovascular system, especially in the heart and blood vessels, modulating a wide variety of physiological processes and participating in disease conditions [19]. In the placenta, ILK expression is critical during the first stages of pregnancy, regulating particular cellular behaviors [20].In addition, ILK seems to play a major role in the development of pre-eclampsia, arising as an important therapeutic target [21]. Cadherins are transmembrane proteins implicated in cell-to-cell adhesion and are central determinants of tissue cytoarchitecture. Epithelial cadherin (e-cadherin) is one of the best-characterized cadherins studied, regulating cell development and morphogenesis from early stages [22] and with adverse consequences in the placenta when dysregulated [23]. Other members of the cadherin family such as cadherin 6 and 17 are also arising as promising indicators of health and disease status [24].

Thus, the purpose of this study is to analyze the differential expression of ILK, e-cadherin, cadherin 6 and cadherin 17 in the placenta of women with CVD in comparison to healthy controls. Gene and protein expression will be detected by real time quantitative PCR (RT-qPCR) and immunohistochemistry, respectively.

## 2. Patients and Methods

### 2.1. Experimental Design

We have performed an observational, analytical and prospective study including 114 women in the third trimester of pregnancy. Of them, there were 62 women diagnosed with CVD according to the CEAP classification [25] and 52 women without a history of CVD, referred as healthy controls (HC). The median age of women with CVD was 33 years (interquartile range (IQR), 22–40 years) and the median gestational period was 40.5 weeks (IQR, 39–41.5 weeks), whereas HC had a median gestational age of 34 years (IQR, 27–41 years) and a median gestational period of 41 weeks (IQR, 39–42 weeks). The current work was completed following the basic ethical principles of autonomy, beneficence, non-maleficence and distributive justice. Furthermore, the regulations of Good Clinical Practice, as well as the principles set forth in the last Declaration of Helsinki (2013) and the Oviedo Convention (1997) were also followed. Patients were informed prior to enrolment, and each participant provided their corresponding written consent. The present study was approved by the Clinical Research Ethics Committee of the Central University Hospital of Defence University of Alcalá (37/17). During the third trimester consultation, the clinical history was reviewed and a general physical examination of the woman was performed. Moreover, lower limb ultrasounds were performed using an Eco-Doppler (Portable M-Turbo Eco-Doppler; SonoSite, Inc., Bothell, WA, USA) at 7.5 MHz.

The inclusion criteria of our study were defined as women > 18 years of age, with clinical evidence of lower limb venous disease during the third trimester, according to CEAP (≥1). On the other hand, the exclusion criteria included women with prior diagnosis of high blood pressure; venous malformations; heart, kidney and lung insufficiency; autoimmune diseases; body mass index ≥ 25; diabetes mellitus, gestational diabetes mellitus or other endocrine diseases; active infectious diseases; toxicological habits (alcohol (≥1 unit a day), tobacco (≥1 cigarette a day), or drugs (e.g., cannabis, heroin, cocaine, amphetamines)); pre-eclampsia and/or HELLP syndrome; known causes of intrauterine growth restrictions; existence of pathological injuries, such as placental infarction, avascular villi, delayed villi maturation or chronic villitis; as well as the appearance of any exclusion criteria in the following months (until delivery); and previous evidence of CVD.

There were no significant differences between the groups regarding the number of previous pregnancies: 33 (53.2%) for women with CVD and 19 (36.5%) for women in the HC group (Table 1). There were also no significant differences in the clinical characteristics between the CVD and HC groups (gestational age, c-section delivery, previous pregnancies, previous abortions, regular menstrual cycles and type of profession-sedentary, Table 1).

### 2.2. Tissue Samples

Placental biopsies were collected after delivery for the 114 patients. In every case, 5 placental fragments were obtained by using a scalpel to include various mixed cotyledons. Then, placental pieces were added to two different sterile tubes: One containing Minimum Essential Medium (MEM; Thermo Fisher Scientific, Inc., Waltham, MA, USA) with 1% antibiotic/antimycotic (Streptomycin, Amphotericin B and Penicillin; Thermo Fisher Scientific, Inc.) and another with RNAlater^®^ (Ambion; Thermo Fisher Scientific, Inc., Waltham, MA, USA) solution. Subsequently, the samples were processed in a class II laminar flow hood (Telstar AV 30/70 Müller 220 V 50 MHz; Telstar; Azbil Corporation) in a sterile environment. Conserved samples were stored in 1 mL RNAlater^®^ at −80 °C until further processing for gene expression analysis. Preserved MEM placentas were employed for histological and immunohistochemical studies.

MEM samples were washed and rehydrated five times in MEM without antibiotics to remove the erythrocytes. After, they were cut into 2 cm fragments and fixed in F13 (60% ethanol, 20% methanol, 7% polyethylene glycol and 13% distilled water) following established protocols [26]. Then, samples were embedded in paraffin, using molds. Once the paraffin had solidified, a HM 350 S rotation microtome (Thermo Fisher Scientific, Inc., Waltham, MA, USA) was used to obtain 5-µm thick sections, which were then stretched in a hot water bath and mounted on glass slides, previously treated with 10% polylysine, in order to enhance adhesion of the sections.

### 2.3. Gene Expression Studies Using Reverse Transcription-Quantitative PCR (RT-qPCR)

RNA was extracted following the guanidinium thiocyanate-phenol-chloroform method [27,28] allowing the analysis of mRNA expression levels of the selected genes. RNA samples at a concentration of 50 ng/µL were used to synthesize complementary DNA (cDNA) by reverse transcription (RT). 4 µL of each sample is mixed with 4 µL of oligo-dT 0.25 µg/µL solution (Thermo Fisher Scientific, Inc., Waltham, MA, USA), and incubated at 65 °C for 10 min in a dry bath (AccuBlock, Labnet International Inc., NJ, USA) to denature the RNA [16,29]. Hereunder, samples were put on ice and 10 µL of a reverse transcription mix containing the following products was added for each sample: 2.8 µL First Strand Buffer 5X (250 mM Tris-HCl and pH 8.3; 375 mM KCl; 15 mM MgCl2) (Thermo Fisher Scientific, Inc., Waltham, MA, USA); 2 µL of 10 mM deoxyribonucleotides triphosphate; 2 µL of 0.1 M dithiothreitol; 1.7 µL of DNase- and RNase-free water; 0.5 µL of RNase inhibitor (RNase Out); 1 µL of reverse transcriptase enzyme (all from Thermo Fisher Scientific, Inc., Waltham, MA, USA).

The RT process was conducted using a G-Storm GS1 thermal cycler (G-Storm Ltd.). Then, the samples were incubated at 37 °C for 75 min in order to allow cDNA synthesis. At this point, the temperature was increased to 70 °C and maintained for 15 min, thereby causing the denaturation of the reverse transcriptase enzyme, and the temperature was gradually reduced to 4 °C. A negative reverse transcription was performed to ensure the absence of genomic DNA contamination in the total RNA samples, in which the M-MLV RT enzyme is replaced by water free of DNases and RNases. The cDNA produced in RT was diluted 1:20 in water free of DNases and RNases and stored at −20 °C until further use.

Specific primers for the selected genes (Table 2) were designed de novo through the Primer-BLAST and AutoDimer online applications [30,31]. The constitutively expressed TATA-box binding protein (TBP) gene was employed as a control to normalize the results [32]. The gene expression units are expressed as relative quantities of mRNA. RT-qPCR was performed on a StepOnePlus™ System (Applied Biosystems; Thermo Fisher Scientific, Inc.) using the relative standard curve method. The reaction was completed as follows: 5 µL sample [mixed at 1:20 with 10 µL iQ™ SYBR^®^ Green Supermix (Bio-Rad Laboratories, Inc.)] was mixed with 1 µL each forward and reverse primers, and 3 µL of DNase and RNase-free water, which were then added to a MicroAmp^®^ 96-well plate (Applied Biosystems; Thermo Fisher Scientific, Inc., Waltham, MA, USA). The following thermocycling conditions were used: Initial denaturation for 10 min at 95 °C, denaturation for 15 s at 95 °C, annealing at variable temperatures depending on the melting temperature of each primer pair for 30 s, and elongation at 72 °C for 1 min, for 40–45 cycles. Then, a dissociation curve for 15 s at 95 °C, 1 min 60 °C, 15 s 95 °C, and 15 s 60 °C was developed. Fluorescence detection was performed at the end of every repeat cycle (amplification) and at the different steps of the dissociation curve. The data collected from the selected genes were included in a standard curve made by serial dilutions of a mixture of the samples, that were included in each plate according to the constitutive expression of TBP (in agreement with the manufacturer’s protocols). This RT-qPCR was performed twice in all samples of placenta tissue.

### 2.4. Immunohistochemistry Studies for Protein Expression Analysis

The antigen/antibody reactions were detected using the avidin-biotin complex method, with avidin-peroxidase, as previously described [33,34]. Immunohistochemical studies were performed on paraffin-embedded placental samples. The antibodies used in our study are described in the protocol specifications (Table 3). The samples were incubated with the primary antibody (90 min; Table 3), and then with 3% BSA Blocker (cat. no. 37,525; Thermo Fisher Scientific, Inc., Waltham, MA, USA) and PBS overnight at 4 °C. The next day, the placental tissues were incubated with biotin-conjugated secondary antibody diluted in PBS, for 90 min at room temperature (Table 3). Afterwards, the avidin-peroxidase conjugate ExtrAvidin^®^-Peroxidase (Sigma-Aldrich; Merck KGaA, San Luis, MO, USA) was added for 60 min at room temperature (1:200 dilution with PBS). Eventually, the protein expression level was determined using a chromogenic diaminobenzidine (DAB) substrate kit (cat. no. SK-4100; Maravai LifeSciences, CA, USA), which was prepared just before exposure (5 mL distilled water; two drops buffer; four drops DAB; and two drops hydrogen peroxide). The signal was developed with the peroxidase chromogenic substrate for 15 min at room temperature, allowing the detection of a brown stain. For each protein, sections of the same tissue were assigned as negative controls, substituting incubation with the primary antibody for a blocking PBS solution. In all tissues, the contrast was achieved using the Carazzi hematoxylin for 15 min.

Preparations were observed using a Zeiss Axiophot optical microscope (Zeiss GmbH). Then, 5 sections and 10 fields of view were randomly examined for each patient of the defined groups. The patients were described as positive when the marked mean area in the analyzed sample was ≥5% of the total, following the immunoreactive score (IRS) as established in previous studies [35,36]. Immunostaining was evaluated by two independent histologists, and then each sample was scored using the following scale: 0–1, minimum staining (≤25%); 2, moderate staining (25–65%); and 3–4, strong staining (≥65–100%).

### 2.5. Statistical Analysis

The statistical analysis was performed using the the GraphPad Prism^®^ v6.0 (GraphPad, Inc., San Diego, CA, USA) program. The Mann–Whitney U test was used to compare the 2 groups, and the data was expressed as the median ± SEM. Significance was established as *p* < 0.05 (*), *p* < 0.01 (**), and *p* < 0.001 (***).

## 3. Results

### 3.1. Women with CVD during Pregnancy Show an Increase in ILK Expression in Placental Villi Associated with a Decrease in E-Cadherin

**Figure 1:**
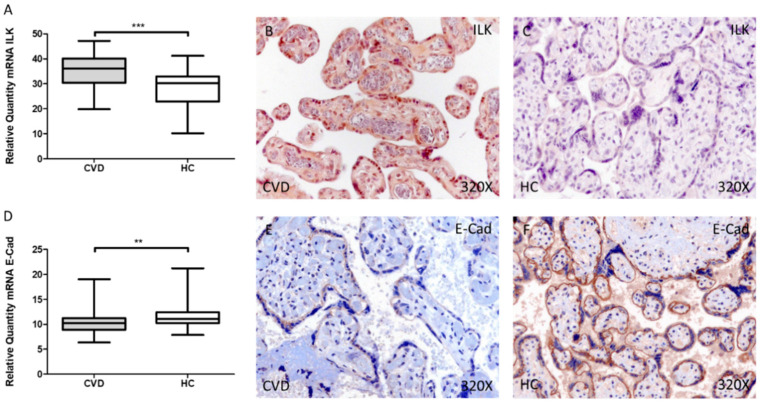
Levels of mRNA for ILK (**A**) and E-Cad (**D**) quantified by RT-qPCR, and histological imágenes for immunohistochemical techniques in the placental villi in of women with CVD during pregnancy and HC for ILK (**B**,**C**) and E-Cad (**E**,**F**). CVD = Chronic venous disease, HC = Healthy control. *p* < 0.01 (**), and *p* < 0.001 (***).

Our results have shown a significant increase in ILK gene expression in the placental villi of women with CVD during pregnancy compared to HC, *** *p* < 0.001 [CVD = 35.027 ± 0.817 vs. HC = 28.388 ± 0.934, Figure 1A]. Histological analysis of protein expression using immunohistochemical techniques showed a significant increase in ILK expression in the placental villi of women with CVD during pregnancy compared to HC, *** *p* < 0.001 [CVD = 2.379 ± 0.084 vs. HC = 0.976 ± 0.082, Figure 1B,C].

Conversely, we observed a significant decrease in E-Cad gene expression in the placental villi of women with CVD during pregnancy compared to HC, ** *p* = 0.0084 [CVD = 10.726 ± 0.359 vs. HC = 11.893 ± 0.461, Figure 1D]. In parallel, protein expression showed a significant decrease in E-Cad expression in the placental villi of women with CVD during pregnancy compared to HC, *** *p* = 0.002 [CVD = 0.903 ± 0.062 vs. HC = 1.240 ± 0.058, Figure 1E,F].

### 3.2. Cadherin 17 and Cadherin 6 Expression Level Is Increased in the Placental Villi of Women with CVD during Pregnancy

**Figure 2:**
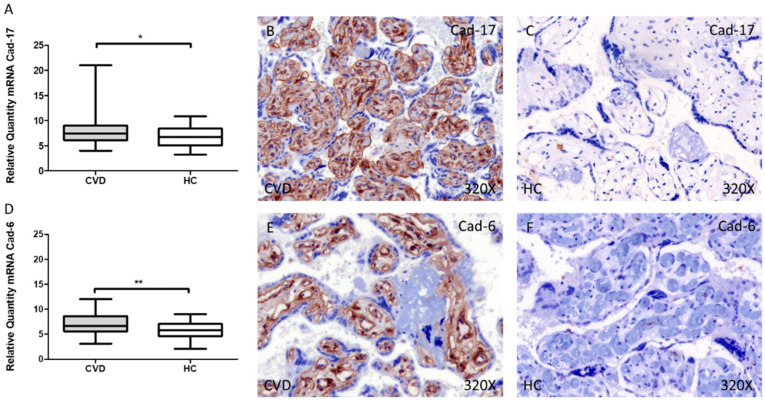
Levels of mRNA for Cad-17 (**A**) and Cad-6 (**D**) quantified by RT-qPCR, and histological imágenes for immunohistochemical techniques in the placental villi in of women with CVD during pregnancy and HC for Cad-17 (**B**,**C**) and Cad-6 (**E**,**F**). CVD = Chronic venous disease, HC = Healthy control. *p* < 0.05 (*), *p* < 0.01 (**).

Cad-17 gene expression showed a significant increase in the placental villi of women with CVD during pregnancy compared to HC, * *p* = 0.0228 [CVD = 7.804 ± 0.325 vs. HC = 6.780 ± 0.263, Figure 2A]. In this sense, protein expression showed a significant elevation using immunohistochemical techniques in the placental villi of women with CVD during pregnancy compared to HC, ** *p* = 0.0026 [CVD = 1.403 ± 0.067 vs. HC = 1.159 ± 0.085, Figure 2B,C].

Similarly, our results have shown an increase in the gene expression of Cad-6 in the placental villi of women with CVD during pregnancy compared to HC, ** *p* = 0.0016 [CVD = 7.083 ± 0.251 vs. HC = 5.807 ± 0.247, Figure 2D]. Furthermore, protein expression showed a significant increase in the placental villi of women with CVD during pregnancy compared to HC, ** *p* = 0.0033 [CVD = 1.202 ± 0.065 vs. HC = 0.923 ± 0.066, Figure 2E,F].

## 4. Discussion

For the first time, we have demonstrated significant changes in tissue expression of critical cell to ECM (ILK) and cell to cell (e-cadherin, cadherin 7 and cadherin-9) components in placentas of women with CVD. These results are in consonance with our previous studies that showed that CVD is associated with a set of changes in the placenta in the ECM [7,16] and cell behavior [13,17].

We detected that ILK is overexpressed in placentas of women with CVD. There are multiple studies reporting the same results, particularly in the field of oncology, promoting cell migration and invasion [37]. In the same manner, ILK is highly expressed in the first trimester of pregnancy, where it seems to stimulate and regulate migration and invasion of cytotrophoblast lines in vivo, which is crucial for the process of placentation [20] However, previous studies have found substantial changes in the expression of ILK under pathological pregnancies such as pre-eclampsia or gestational diabetes [38,39]. Similarly, we found a possible role of ILK in the pathophysiology of CVD in the placenta of pregnant women. Within the cell, ILK is crucial for anchoring the actin filaments (microfilaments) to the integrins, also mediating signal transduction between intracellular and extracellular compartments [18]. Regarding the mediation of ILK in extracellular signaling, it is widely accepted that ILK is located in the cell-matrix focal adhesions but not in cell-to-cell adhesion sites [40]. Thus, ILK is essential for the formation of focal complexes and actine anchoring, being also involved in the assembly of fibronectin-bound fibrillar adhesions [41]. This role of ILK appears to be critical for cell cycle regulation, acting through the activation of key proteins involved in cell division like cyclin d1/d3, cyclin-dependent kinase 2/4 (CDK2/CDK4) and the downregulation of the cell cycle inhibitor p27 [42]. Conversely, ILK upregulation induces an anchorage-independent cell cycle progression, resulting in an increased expression of cyclin D1, activation of Cdk4 and an altered p27 expression, eventually leading to the stimulation of G1/S cyclin-Cdk activities, regulated by cell adhesion and integrins in normal conditions [43]. In accordance with this statement, we have previously identified an increased expression of cyclin D1 in the placenta of women with CVD [17] which may be associated with the overexpression of ILK, also indicating an abnormal regulation and progression of the cell cycle in an adhesion-independent manner. On the other hand, ILK is also related to other cell signaling routes. For instance, PI3K/Akt is one of the main signaling routes related to ILK [44]. It seems that ILK is activated in a PI3K-dependent manner and in turn, ILK activates Akt, also suppressing Glycogen synthase kinase 3β (GSK-3β), an inhibitor of c-Jun and β-catenin [45]. Similarly, the PI3K/Akt inhibitor PTEN is also involved in the downregulation of ILK [46]. In agreement with these facts, we have demonstrated that the placenta tissue of women with CVD present an increased expression of PI3K/Akt and β catenin [17] and ILK may play a key role in the activation of these components. In addition, ILK also seems to be a crucial regulator of metalloproteinases (MMPs) particularly MMP-9 [47]. MMP-9 is involved in the remodeling of the ECM and, consistently, we have previously reported an increased expression of this protein in the placenta of women with CVD [7].

Likewise, we have observed a reduced expression of e-cadherin, cadherin 17 and cadherin 6 associated to CVD. Cadherins and prominently, e-cadherin are critical components for maintaining cell attachment and the layered phenotype of the villous cytotrophoblast. On the other hand, reduced expression of cadherins is involved in the loss of cellular connectivity with a reduced apico-basal polarity [48]. Of the different cadherins analyzed in this work, e-cadherin is the most relevant and broadly studied. Prior research shows significant alterations in the expression of e-cadherin in obstetric complications, including in placenta percreta [49], placenta accrete [50], gestational trophoblastic diseases [51] and pre-eclampsia [22]. A reduced expression of e-cadherin seems to be associated with an invasive phenotype and abnormal behavior of the placental cells, hence supporting the possible pathophysiological implications of this component in CVD. A plausible explanation of the reduced expression of e-cadherin in the placenta tissue may be related to the inhibitory action of ILK through the Poly (ADP-ribose) polymerase (PARP) [52]. We have recognized that not only ILK but also PARP are upregulated in the placenta tissue of women with CVD [14] and it is possible that this mechanism explains in part the downregulation of e-cadherin.

Finally, less data are collected regarding the role of cadherin-6 and cadherin-17 in the placenta. Cadherin-6 has been identified as a pivotal molecule implicated in implantation and placentation [53,54]. On the other hand, cadherin-17 is a molecule exclusively expressed in the embryonic epithelial cells and in some parts of the adult gastrointestinal tract [55]. Our results show a significant increase of both cadherins in the placenta of women with CVD. Previous studies have described a pathological association between upregulated cadherin-6 and cadherin-17 with an increased invasiveness and cell proliferation, respectively [24]. To our knowledge, this is the first study to demonstrate a pathophysiological role of cadherin-6 and cadherin-17 in the vascular disorders affecting the placenta. Future studies should be conducted to unravel possible causes and consequences of this dysregulation. Biochemical studies are needed that allow giving more mechanistic information, such as the Immunoblottting method and cultured placental villous explants with inhibitors of ILK or cadherin to better investigate the mechanism.

## 5. Conclusions

Our study demonstrates a significant increase in the protein and gene expression of ILK, cadherin-6 and cadherin-17 and a reduction of e-cadherin, associated to the development of CVD during pregnancy. Consistent with previous results, we demonstrate an abnormal functioning of the placenta in women affected by this condition, probably with negative pathophysiological implications. Future studies should be conducted in order to assess the impact of CVD in maternofetal structures and well-being.

## Figures and Tables

**Table 1:** Clinical and demographic characteristics. CVD = Chronic venous disease, HC = Healthy control.

	CVD (*n* = 62)	HC (*n* = 52)
Median age (IQR), years	33 (22–40)	34 (27–41)
Median gestational age (IQR), weeks	40.5 (39–41.5)	41 (39–42)
C-section delivery, n (%)	12 (19.4)	9 (17.3)
Vaginal delivery, n (%)	50 (80.6)	43 (82.7)
Varicose vein (CEAP), n (%)		
CEAP 1	37 (59.7)	0 (0)
CEAP 2	21 (33.8)	0 (0)
CEAP 3	4 (6.5)	0 (0)
Previous pregnancies, n (%)	33 (53.2)	19 (36.5)
Previous abortions, n (%)	14 (22.6)	9 (17.3)
Regular menstrual cycles, n (%)	50 (80.6)	42 (80.7)
Sedentary profession, n (%)	41 (66.1)	40 (76.9)

**Table 2:** Primer sequences used in RT-qPCR and temperature (Tm).

GENE	SEQUENCE Fwd (5′→3′)	SEQUENCE Rev (5′→3′)	Temp
TBP	TGCACAGGAGCCAAGAGTGAA	CACATCACAGCTCCCCACCA	60 °C
ILK	TCCCAAGTAAGGAACGGAGC	CACCACCAGACATGAGCACT	59 °C
E-Cad	GTGAACACCTACAATGCCGC	CCCAGGGGACAAGGGTATGA	59 °C
Cad-17	GCTCCTGGGAGGTAAGTAGA	ACCCTCGGCAAAGCTCC	57 °C
Cad-6	AGCTATTTCCTGCTTTCAGGGT	GGTGGGAAGGAAGTGAGACG	60 °C

**Table 3:** Primary and secondary antibodies used in the immunohistochemical studies performed, showing the dilutions used and the protocol specifications.

Antigen	Species	Dilution	Provider	Protocol Specifications
ILK	Rabbit	1:50	Abcam (ab52,480)	10 mM Sodium citrate pH = 6 before incubation with blocking solution
E-Cad	Mouse	1:250	Vitro (MAD-000761QD-3/V)	-
Cad-17	Rabbit	1:250	Vitro (MAD-000737QD-3/V)	-
Cad-6	Mouse	1:250	Vitro (MAD-000582QD-3/V)	-
IgG (Mouse)	Goat	1:300	Sigma-Aldrich (F2012/045K6072)	-
IgG (Rabbit)	Mouse	1:1000	Sigma-Aldrich (RG-96/B5283)	-

## Data Availability

The data used to support the findings of the present study are available from the corresponding author upon request.
